# Persistent postseptoplasty nasal neuropathic pain

**DOI:** 10.1097/PR9.0000000000001266

**Published:** 2025-04-03

**Authors:** Gio Gemelga, Shweta Chawla, Shikha Sharma, Xiaobing Yu

**Affiliations:** a School of Medicine, University of California, San Francisco, San Francisco, CA, USA. Gemelga is now with the Department of Anesthesia, St. Joseph's Medical Center, Stockton, CA, USA; b Center for Pain Medicine, Department of Anesthesia and Perioperative Care, University of California, San Francisco, San Francisco, CA, USA

## *Letter to the Editor:*

Estimated to exceed a quarter million procedures annually,^5^ nasal septoplasty is the most common otolaryngology operation in the United States. The effectiveness of septoplasty in improving nasal obstruction has been well studied^1,5^; chronic postsurgical complications have long been thought to be extremely rare.^2^ To our surprise, we now identified a cohort of patients with persistent postseptoplasty nasal pain, an unreported complication in the surgical literature. Thus, a subsequent observational study was conducted to determine the demographic characteristics and associated risk factors.

**Figure 1.**
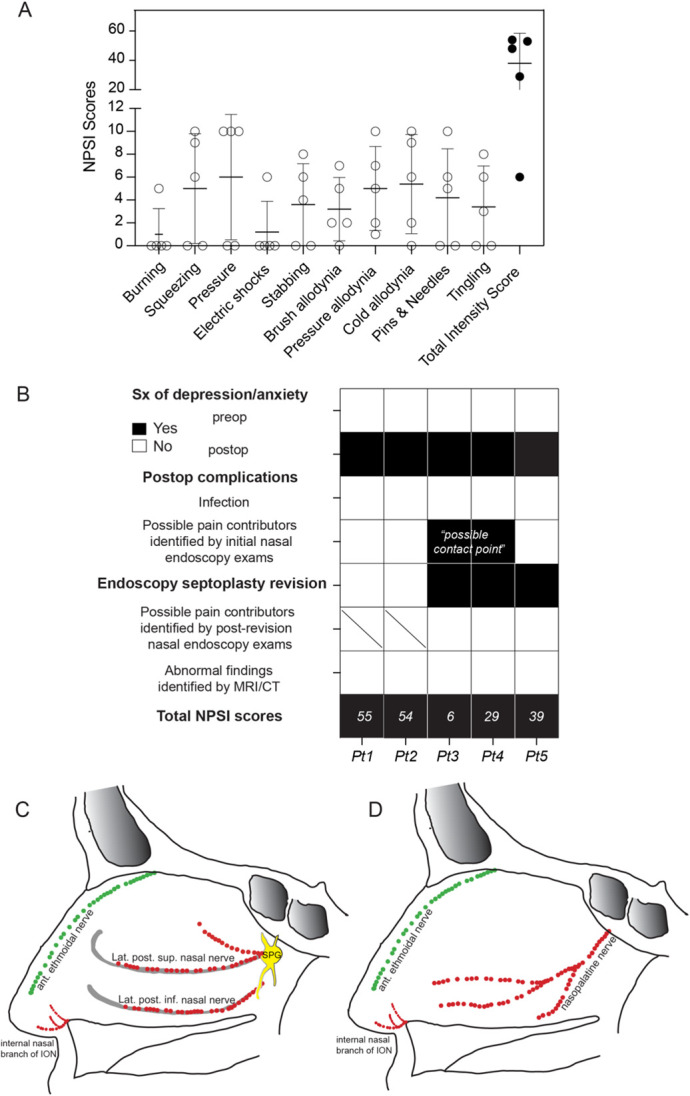
Assessment of neuropathic pain symptoms and neuroanatomical pain distribution. (A) Summary of neuropathic pain symptom profiling in a cohort of 5 patients with persistent postseptoplasty nasal pain. Data presented as mean ± SD. (B) Evaluation of potential underlying psychological and anatomical pain contributors. (C–D) Schematic diagram of the major sensory innervation of the nose. The major sensory nerve supply to the lateral nasal wall (C) and nasal septum (D) is derived from the maxillary (V2, red) and ophthalmic divisions (V1, green) of trigeminal nerve. ant., anterior; inf., inferior; ION, infraorbital nerve; lat., lateral; NPSI, neuropathic pain symptom inventory; post., posterior; postop, postoperative; preop, preoperative; Pt, Patient; SPG, sphenopalatine ganglion; sup., superior; Sx, Symptoms.

Our cohort included 5 otherwise healthy patients who developed persistent nasal pain immediately after uncomplicated endoscopic septoplasty to correct nasal obstruction and septal deviation (Table 1). Neither underlying psychological nor definitive anatomical pain contributors were identified (Fig. 1). Using the Neuropathic Pain Symptom Inventory, a validated neuropathic pain self-assessment questionnaire, we confirmed that all patients had positive sensory abnormalities (eg, spontaneous pain, allodynia to tactile and cold stimuli, and pins & needles), the hallmark of neuropathic pain (Fig. 1). Notably, all were young Caucasian male patients, with a mean (median) age of 26.6 (24) years at the diagnosis of nasal allodynia. Importantly, inferred from history and physical examination, the distribution of pain was mediated by the ophthalmic (V1) branch of the trigeminal nerve in 2 patients, the maxillary (V2) branch in 2 patients, and both V1 and V2 in 1 patient (Fig. 1). Pain innervation was further confirmed with selective trigeminal nerve blocks in 4 patients. After the updated NeuPSIG grading,^3^ we determined that neuropathic pain was “probable” in 1 patient and “definite” in 4 patients (Table 1).

**Table 1:** Demographic and clinical characteristics.

Clinical characteristics	Statistics
Ethnicity	
Caucasian	5/5 (100%)
Gender	
Male	5/5 (100%)
Current age (y)	
Mean ± SD	31.8 ± 5.7
Median (min, max)	33 (26, 39)
Age at diagnosis (y)	
Mean ± SD	26.6 ± 2.6
Median (min, max)	24 (19, 35)
Surgical indications	
Nasal obstruction	5/5 (100%)
Deviated septum	5/5 (100%)
Surgical history	
Endoscopic septoplasty	5/5 (100%)
Endoscopic septoplasty revision	3/5 (60%)
Medical history	
Chronic pain before surgery	0/5 (0%)
Neuropathic pain features	
Mechanical allodynia	4/5 (80%)
Thermal allodynia	2/5 (40%)
Nasal pain innervation	
V1	2/5 (40%)
V2	2/5 (40%)
V1 + V2	1/5 (20%)
Confirmed with selective nerve block (s)	4/5 (80%)
Postsurgical imaging via computed tomography/magnetic resonance imaging	
Unremarkable finings	5/5 (100%)
Extent of pain disability	
Unable to work	3/5 (60%)
Multimodal pharmacologic pain treatment	
Non-steroidal anti-inflammatory drugs, acetaminophen	5/5 (100%)
Anticonvulsants	
Gabapentinoids	5/5 (100%)
Sodium channel blockers	5/5 (100%)
Antidepressants	
Tricyclics (TCAs)	4/5 (80%)
Serotonin–norepinephrine reuptake inhibitors (SNRIs)	4/5 (80%)
Opioids	4/5 (80%)
Low-dose ketamine infusion	2/5 (40%)
Pain classification using the updated NeuPSIG grading^3^	
Complaint of persistent postsurgical pain	5/5 (100%)
History of relevant neurological lesion and pain distribution neuroanatomically plausible	5/5 (100%)
Pain is associated with sensory signs in the same neuroanatomically plausible distribution on examination	5/5 (100%)
Diagnostic test confirming a potential lesion of the somatosensory nervous system explaining the pain	4/5 (80%)
Definite neuropathic pain	4/5 (80%)
Probable neuropathic pain	1/5 (20%)

In all 5 patients, nasal neuropathic pain was recalcitrant to multimodal pharmacological treatment (Table 1). Moreover, 3 patients even had septoplasty revision without benefit. Given that nasal allodynia was innervated by the trigeminal nerve, we conducted radiofrequency ablation of the V2 branch in 2 patients, including 1 who had a revision, with resolution of their pain symptoms.^4^

In this article, we demonstrate that nasal neuropathic pain is likely an underestimated chronic complication of septoplasty. The major sensory innervation of the nose is comprised of a combination of the V1 and V2 divisions^4^ (Fig. 1), which can be injured during surgical manipulation. Our findings further support that targeting the associated trigeminal nerve in the patient's pain distribution can be considered before revision is recommended.

Notably, only a small number of patients were assessed at a single tertiary care center. Although 30% of patients undergoing septoplasty are women,^1,5^ our cohort consisted of all young Caucasian male patients. Future research is needed to confirm the generalizability, particularly in ethnically diverse populations. Nevertheless, it is important for clinicians to recognize this underestimated complication which can profoundly affect the well-being of young adults.

## Disclosures

The authors have no conflict of interest to declare.
